# Rethinking MRI as a measurement device through modular and portable pipelines

**DOI:** 10.1007/s10334-025-01245-3

**Published:** 2025-04-24

**Authors:** Agah Karakuzu, Nadia Blostein, Alex Valcourt Caron, Arnaud Boré, François Rheault, Maxime Descoteaux, Nikola Stikov

**Affiliations:** 1 NeuroPoly Lab, Polytechnique Montreal, Montreal, Québec Canada; 2 Montreal Heart Institute, University of Montreal, Montreal, Québec Canada; 3 Sherbrooke Connectivity Imaging Laboratory (SCIL), Computer Science Department, Université de Sherbrooke, Sherbrooke, Québec Canada; 4 Center for Advanced Interdisciplinary Research, Ss. Cyril and Methodius University, Skopje, North Macedonia; 5 NYUAD Research Institute, New York University Abu Dhabi, Abu Dhabi, UAE; 6 School of Medicine, University Collage Cork, Cork, Ireland

**Keywords:** MRI workflows, Reproducibility, Standardization, Metrology, Vendor-neutral, Quantitative MRI

## Abstract

The premise of MRI as a reliable measurement device is limited by proprietary barriers and inconsistent implementations, which prevent the establishment of measurement uncertainties. As a result, biomedical studies that rely on these methods are plagued by systematic variance, undermining the perceived promise of quantitative imaging biomarkers (QIBs) and hindering their clinical translation. This review explores the added value of open-source measurement pipelines in minimizing variability sources that would otherwise remain unknown. First, we introduce a tiered benchmarking framework (from black-box to glass-box) that exposes how opacity at different workflow stages propagates measurement uncertainty. Second, we provide a concise glossary to promote consistent terminology for strategies that enhance reproducibility before acquisition or enable valid post-hoc pooling of QIBs. Building on this foundation, we present two illustrative measurement workflows that decouple workflow logic from the orchestration of computational processes in an MRI measurement pipeline, rooted in the core principles of modularity and portability. Designed as accessible entry points for implementation, these examples serve as practical guides, helping users adapt the frameworks to their specific needs and facilitating collaboration. Through critical evaluation of existing approaches, we discuss how standardized workflows can help identify outstanding challenges in translating glass-box frameworks into clinical scanner environments. Ultimately, achieving this goal will require coordinated efforts from QIB developers, regulators, industry partners, and clinicians alike.

## Introduction

*“The principal task of a measurement system is the sensing of the measured value of a physical quantity or of a measurement signal representing the wanted measured value.”*

Basics of measurement technology—Part 2: Terms for measuring instruments [1].

When evaluated against this standard, MRI scanners are not considered true measurement systems. Their intended use lacks the metrological rigor required for quantitative applications in clinical settings [2, 3]. As such, they are clinical tools designed for qualitative imaging, not for quantitative science. Nevertheless, measurements derived from MRI are integral to most research in the field.

Quantitative imaging biomarkers (QIBs) are numerical characteristics derived from quantitative imaging techniques [4]. However, specific definitions of MRI measurements depend on context. For instance, quantitative MRI (qMRI) can measure the relaxation time constants T1 and T2 (in seconds). Such *physics-based QIBs* are obtained by producing systematic variations in voxel values, which can then be fitted to a signal representation. Translating complex tissue properties into measurable QIBs necessitates a more layered modeling approach [5], such as the quantification of white matter microstructure with diffusion MRI (dMRI) [6], or g-ratio by combining dMRI, magnetization transfer or relaxometry imaging [7].

On the other hand, the measurement concept goes beyond purely quantitative frameworks, incorporating quantifiable features extracted from morphological characteristics in imaging data. These *data-driven QIBs* are generated by computational image analysis methods, such as radiomic feature extraction [8, 9] using conventional processing or deep learning [10]. For example, tumor volume (mm^3^) qualifies as a data-driven QIB [4].

All of these measurement procedures ultimately boil down to three main methodological steps: pulse sequence implementations (*Acq*), image reconstruction algorithms (*Recon*), and downstream processing methods (*Post*). For the rest of this article, we use *workflow* for the overall process and *pipeline* for the software that automates it.

Setting aside the impact of inevitable variations in experimental conditions (e.g., hardware differences) and random factors, the generalizability of these three primary steps (*Acq, Recon, and Post*) is crucial to the success of a QIB. Nevertheless, most MRI software development and execution environments are segregated by vendor-specific boundaries, which makes it a challenge to control these three main methodological steps across various experimental settings. In addition to triggering the well-known *“but it works on my machine!”* syndrome, the isolated and proprietary nature of commercial MRI development environments often hinders reproducibility and collaboration between imaging sites. This fragmentation creates significant barriers to innovation and hampers the consistency of QIBs in research and clinical applications.

For example, a recent editorial on radiomics identified this issue as the elephant in the scanner room that must be addressed for data-driven QIBs [11]. Authors emphasized that trustworthy radiomic features should not be influenced by the texture variations of different scanners, to the point where one could infer the vendor based on the images. The inevitable capture of these patterns by deep learning applications clearly impacts the performance and generalizability of the developed models trained on multicenter data, primarily due to a phenomenon known as domain shift [12]. To compensate for such effects when multiple large-scale datasets are pooled in search of true biological variability, statistics or deep learning based data harmonization methods [13] for normative modeling [14] have become increasingly popular.

Nevertheless, this drawback is not unique to data-driven QIBs. For nearly 50 years, physics-based QIBs have been suggested as a powerful alternative to improve the consistency of qualitative pattern recognition (e.g., using T2 to eliminate inter-site contrast variations in T2-weighted images due to protocol differences [15]) and to bring about a whole new era of precision MRI (e.g., leveraging T2 values themselves to establish normative thresholds for routine diagnostics [16]). However, this aspirational vision for physics-based QIBs—that they might serve as universal scalars to ease radiological interpretations across centers and/or as reliable tools to convert such interpretations into an objective numbers game—remains out of reach.

The past two decades in MRI research have seen synergistic forces to lift QIBs from the swamp of variability: grassroots open-source development [17–19] and more concerted efforts like the Quantitative Imaging Biomarkers Alliance (QIBA), which united, clinicians, industry partners, and regulatory scientists with the mission of reducing variability from QIBs [20]. While independent groups create open-source solutions targeting specific workflow steps, QIBA established profile stages that evaluate a QIB’s ability to support its stated claim across four levels of confidence [21]. As of today, only MR elastography of the liver [22] and the apparent diffusion coefficient [23] are listed as level three (clinically feasible), while cerebral blood volume [24], T1_p_ and T2 for cartilage [25], and dynamic contrast enhancement quantification [26] are designated as level two (consensus) profiles. These are promising developments that have emerged over the past five years. Undoubtedly, the next iterations of QIBA [27] will continue to build on its previous successes.

**Fig. 1:**
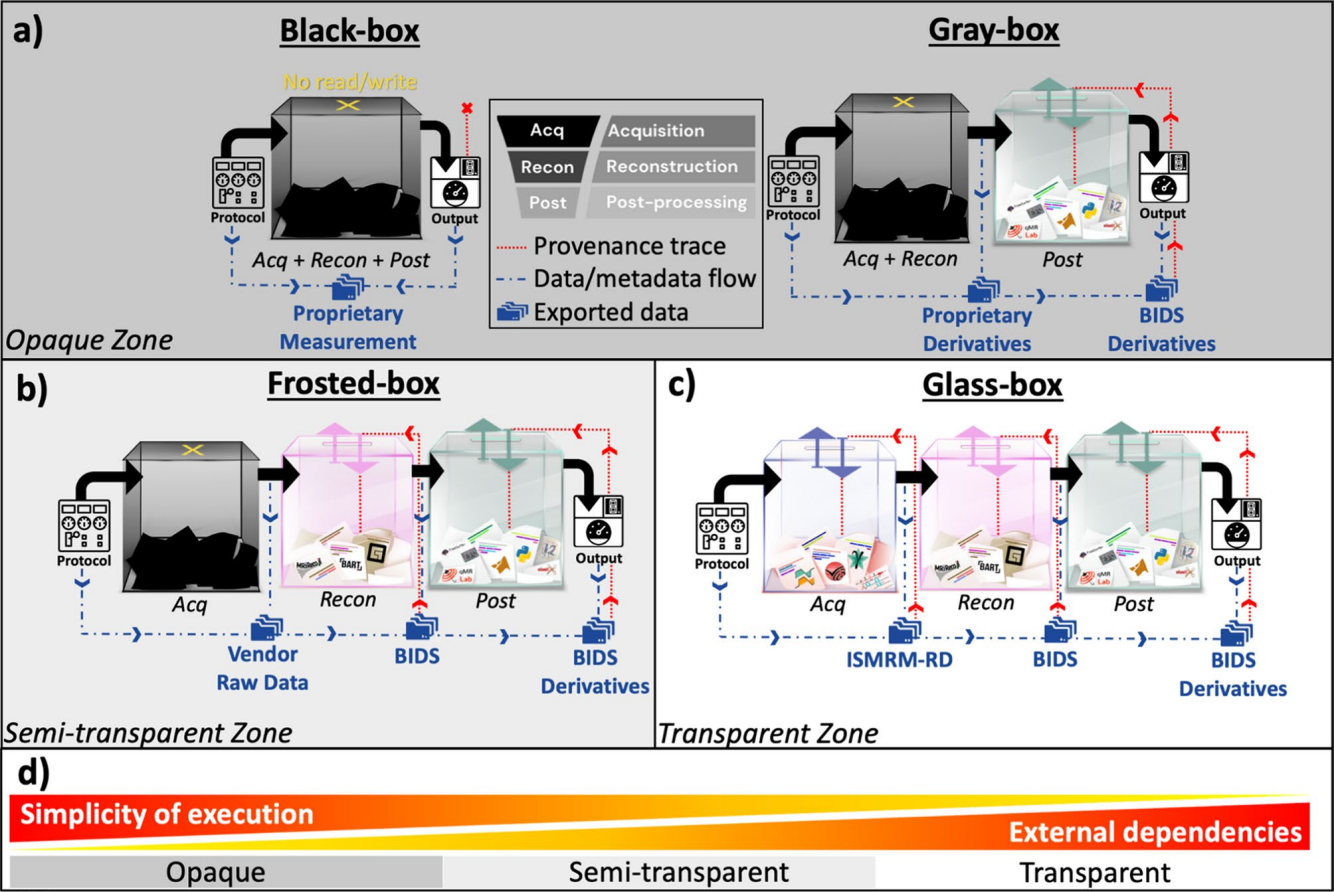
Benchmarking of MRI measurement workflows based on the openness of their key steps: acquisition (*Acq*), reconstruction (*Recon*), and downstream post-processing (*Post*). The extent of traceability (red dashed lines) of the generated data flow (blue dashed lines) are indicated along with the preferred use of community data standards, wherever possible. **a** The opaque zone encompasses black- and gray-box workflows, where *Acq* and *Recon* steps are hindered. **b** The semi-transparent zone corresponds to frosted-box workflows, which limit openness at the *Acq* step. **c** The transparent zone requires glass-box workflows, ensuring full openness across all MRI measurement steps. **d** Variations in execution simplicity and external dependencies across transparency zones

**Fig. 2:**
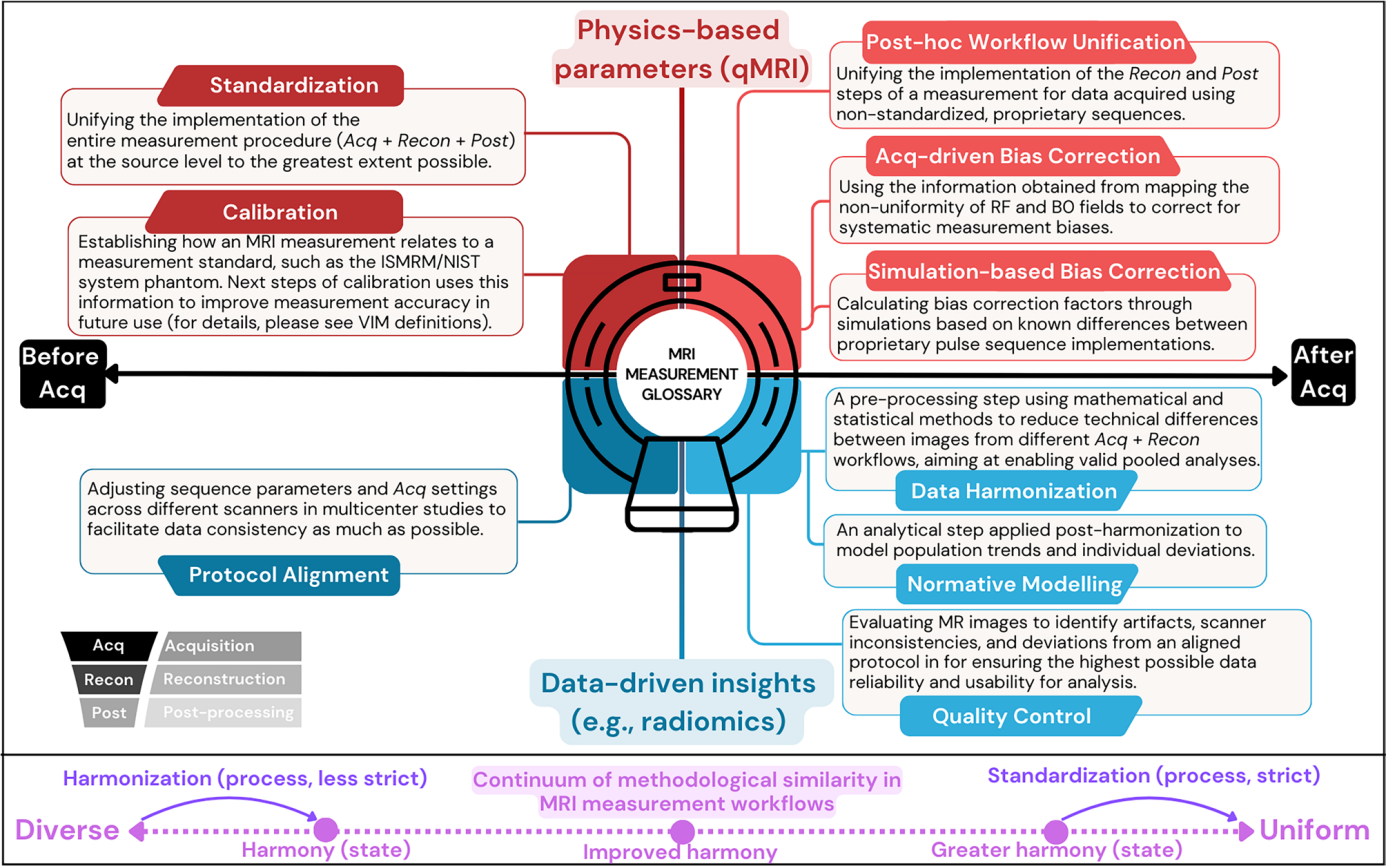
Infographic defining MRI measurement terms used consistently throughout this review. The horizontal axis separates terms based on their relevance before or after acquisition (k-space sampling), while the vertical axis indicates whether their use is geared towards physics-based parameter quantification (i.e., qMRI) or data-driven insights (i.e., radiomics). The continuum of methodological similarity in MRI measurement workflows, based on the definitions by [48]: standardization involves the unification of all steps (*Acq*, *Recon*, and *Post*) to converge towards uniformity, whereas harmonization aims at moving away from total diversity to achieve better harmony. Please refer to VIM and Kessler et al. [4] for a more comprehensive list of terms relevant to MRI measurements [4, 47]

The momentum of open-source community initiatives is a powerful driver in advancing QIBs. To leverage this momentum effectively, we must implement overarching strategies throughout the development process. In doing so, respecting the boundaries of regulatory requirements and commercial interests is key to landing innovations in clinical practice. Playfully captured in the ISMRM 2024 Lauterbur Lecture by Andrew Webb [28], *a chicken-egg dilemma* arises from the conflict between the open-science hype and skepticism. This debate playfully highlights the need to balance enthusiasm for open science with the caution required to tackle its practical challenges.We believe the first step to achieving this balance is to clearly determine how open a QIB must be for its claim to hold. To this end, we introduce a tiered benchmarking framework for MRI measurement workflows by borrowing the concepts of software testing implementations [29]: black-box, gray-box, frosted-box, and glass-box (Fig. 1). The next crucial need is clearly communicating the methods implemented to reduce the variability from MRI measurements. To support this, we have created a mini glossary to foster consistent use of relevant terminology (Fig. 2). As an overarching strategy for creating interoperable, modular, and reproducible measurement pipelines that can propel QIBs through levels of confidence, we propose the use of NextFlow [30] in tandem with well-maintained containers and community data standards. We also provide two example pipelines to illustrate this approach and further discuss implementation challenges and the latest solutions for bringing QIBs closer to practical use.

## Benchmarking the openness of MRI measurement workflows

MRI workflows can be categorized into four benchmarks distributed across three zones of transparency:

- Opaque zone: When the internal implementations of MRI workflows (*Acq *and *Recon*) are unknown and inaccessible (i.e., no read/write access).
  - Black-box benchmark: No visibility into any key step.
  - Gray-box benchmark: Provides access to *Post* but remains closed at earlier stages (*Acq* and *Recon*).
- Semi-Transparent zone (Fig. 1b): A vendor-native acquisition followed by open-source *Recon* and *Post*.
  - Frosted-box benchmark: This partial transparency allows limited modifications to on-site steps of an MRI measurement.
- Transparent zone: (Fig. 1c): When all implementations (*Acq*, *Recon*, and *Post*) are fully accessible for both viewing and editing.
  - Glass-box benchmark: Represents the ideal case of full visibility and control over MRI workflows.

## Making sense of numbers within the opaque zone of MRI measurements

Freed from the complexity of setting up third-party execution environments and dependencies at the scanner site, black-box workflows are often the most user-friendly choice (Fig. 1d). They offer turnkey measurements whose complexities are isolated from the end users by proprietary barriers. An alternative to this monolithic architecture is offered by typical gray-box workflows that offload *Post* tasks to a remote server to extract measurements from vendor reconstructed images. When equipped with advanced data management and off-site network solutions, gray-box measurement workflows can also remove the complexity away from the end user’s experience. Nevertheless, these conveniences come at the expense of dialing into the implementation details to identify potential sources of measurement bias.

*Case*-1: A notable black-box example is the comparison of cardiac T1 maps produced by the proprietary implementations of the MOLLI, shMOLLI, and SASHA sequences [31]. Reliable observation of the T1 changes would help quantify the severity of conditions such as myocardial iron overload (lower T1) and edema (higher T1) [32]. However, when the measurement bias between different methods (or between implementations of the same method across vendors) exceeds the differences observed between diagnostic groups, the clinical utility of the measurement becomes compromised.

Other common examples of commercially available quantification packages are fraction measurement methods, typically used for investigating musculoskeletal and hepatic anomalies [33, 34]. As noted by Cashmore et al. (2021), the main issue with such proprietary packages is their infeasibility for modeling uncertainty propagation (through *Acq*, *Recon*, and *Post*), rendering it impractical to determine measurement uncertainty [2].

*Case*-2: Notably, the lack of inter-vendor agreement in MRI measurements is not specific to a single domain and black-box workflows. Similar challenges arise in other gray-box benchmarks, such as whole-brain T2 mapping using dual-echo FSE data from the ADNI dataset [35], even when *protocol alignment* and phantom-based *quality control* (Fig. 2) are carried out in a highly regimented manner [36].

This shortcoming remains evident across a spectrum of measurements, from the most fundamental T1 mapping methods [37, 38] to fractional anisotropy (FA) in diffusion tensor imaging (DTI) [39], and more sophisticated microstructure imaging approaches [40]. While these examples illustrate the issue, they are far from exhaustive, as many other cases follow a similar pattern [41].

Under more carefully controlled multi-site conditions managed by the same research group, the reproducibility of gray-box workflows can be improved. A notable example is proton-density fat fraction, where each major vendor’s implementation of chemical-shift encoded spoiled gradient echo was used, followed by a standardized *Post* procedure [42]. A similar outcome was also apparent from a global reproducibility challenge for a gold-standard T1 mapping method [37] and MR fingerprinting [43].

Another common roadblock researchers encounter is the challenge posed by proprietary system upgrades. These upgrades may introduce variability that is difficult to account for, even within the same site on the same MRI system [44]. For example, it has been shown that the T1 parameter varies as much as 30% after a scanner upgrade, and a large part of this variation can be attributed to B1 effects. However, B1 calibration is often done at the prescan level, and changes in the RF transmit gain are rarely disclosed by the vendors. The combination of all these factors make the selection of a proper bias correction method a challenge of its own [45]. Note that there is also the impact of variations from the *Acq, Recon,* and *Post* steps. Even with full transparency, these steps may not protect the measurement from variations arising from deeper-level system modifications that the acquisition controllers depend on [46].

## A glossary for navigating the zones of MRI measurements

Correcting for the non-biological variance commonly observed in MRI measurements is a non-trivial effort—one that has taken on a life of its own, giving rise to a nomenclature that is increasingly intricate and sometimes difficult to parse. Therefore, we provide a glossary of terms (Fig. 2) and use them consistently throughout this review to clearly distinguish between different approaches striving for consistency in MRI measurements.

It is important to note that both QIBA and the International Vocabulary of Metrology (VIM) offer comprehensive terminology relevant to measurements [4, 47]. Rather than redefining these established terms, our glossary builds upon them, providing additional clarification within the specific context of this review.

The terms standardization and harmonization are used rather loosely by MRI and neuroimaging researchers to refer to the process of “removing scanner effects” either before or after data collection. Even though these two terms are closely related, they actually represent distinct concepts. In the field of metrology, harmonization is an overarching term used to describe ensuring consistency of values measured by different methods [49]. On the other hand, standardization is a more formal and rigorous approach to harmonization, where consistency is assured by making sure that different machines measure the same quantity in the same way, using reference measurement procedures and objects [50]. Even in fields unrelated to MRI and measurement, a similar distinction applies: harmonization is the process of moving away from total diversity of practice (i.e., achieving harmony by reducing variance), while standardization describes a movement toward uniformity (i.e., the removal of variance at its source) [48, 51].

The definitions outlined in Fig. 2 are closely linked to their intended applications—whether physics-based or data-driven measurements—as well as the timing of their use relative to data acquisition (i.e., pre- or post-acquisition). In the subsequent sections of this review, we illustrate each term from Fig. 2 with practical use cases organized according to these classifications.

## Opening up the implementations, that’s how the light gets in

In earlier sections, we presented two examples—Case-1 for cardiac T1 mapping and Case-2 for brain T2 mapping—that highlight the challenges of operating in the opaque zone of MRI measurements. In this section, we introduce two approaches taken by independent research groups to address these challenges and highlight additional efforts that tackle this issue at various levels.

*Case*-1: Open-MOLLI introduced a glass-box solution that enabled informed comparisons of this specific cardiac T1 measurement procedure across different scanners and vendors [52]. Through *standardization* (Fig. 2), Gaspar et al. (2024) demonstrated improved within-site repeatability, while providing a foundation for further advancements in multi-site studies and enhancing the reliability of MOLLI. Furthermore, the open-source availability of Open-MOLLI nucleates the development of other cardiac T1 mapping methods—such as SASHA and shMOLLI—on any vendor’s system, given that the sequence implementations share similar patterns for sampling the longitudinal relaxation recovery curve.

Standardizing MRI measurement workflows through glass-box implementations provides the state-of-the-art approach for systematically comparing measurement procedures across different systems. This standardization can even be leveraged to evaluate the performance of systems expected to operate identically. Keenan et al. (2025) demonstrated this by deploying a standardized relaxometry workflow on both a commercial low-field 0.55 T system and a prototype system ramped down from 1.5 T to 0.55 T [53]. Their findings showed no significant differences between the prototype and commercial systems for the tested relaxometry measurements, offering a powerful example of the confidence that standardized measurement workflows can provide.

In addition to enabling multi-center collaboration and providing generalizable templates that help MRI researchers build on each other’s work [54], standardized workflows significantly enhance inter-vendor agreement in measurements. The first evidence in support of this claim is presented by Karakuzu et al. (2022), reporting up to 23% reduction of inter-vendor bias for the MTsat [55] multiparametric mapping method [56]. Even though this was achieved using a proprietary vendor-neutral operating system [57], following standardized workflows developed using Pulseq [58] demonstrated similar effectiveness. For a standardized single-shell diffusion MRI workflow, a nearly 2.5 fold reduction of standard error in diffusion indices was reported using Pulseq [59], building on the initial efforts by Nunes et al. (2020) that made the sequence implementation available [60]. Within the same reproducible framework, the application of time-division multiplexing led to more accurate relaxometry and diffusion measurements [61]. More recently, extending the application of Pulseq-based diffusion imaging to cardiac studies has led to significant SNR gains across scanners from different vendors [62].

*Case*-2: Even when the implementation cannot be opened up, obtaining some information on the precise timing and component attributes of a pulse sequence can be valuable. Fortunately, vendors may be willing to cooperate with researchers to disclose such information within the framework of their legal guidelines. With these priors, differences between the nominal and actual (physical) cross-vendor effects can be identified by devising mathematical relationships to derive respective correction factors. In addressing Case-2, a successful implementation of this strategy is provided by Chehetri et al. (2021) using Bloch modeling for retrospective correction of T2 bias in the ADNI dataset [63]. In this particular case, the *simulation-based bias correction* (Fig. 2) was performed within the gray-box benchmark, given that the source dataset is reconstructed in a proprietary pipeline.

Similarly, Rowley et al. (2021) developed a simulation framework that models the impact of magnetization transfer pulses, which vary between vendors, for sequence-informed removal of B1 effects from the MTsat maps [64]. Another gray-box bias correction method aimed to control these saturation effects at the sequence implementation level, guided by simulation-based insights [65]. Other examples of simulation-based bias correction include [66–68]. A more common approach to post-hoc correction of systematic bias is acquiring field maps, adding yet another layer of MRI measurement [69]. Consequently, field maps used for *Acq-driven bias correction* (Fig. 2) are subject to the same limitations as QIBs. Lee et al. [70] demonstrated that, despite their corrective intent, different B1^+^ mapping implementations can actually exacerbate inter-vendor variability in T1 measurements [70]. Nevertheless, B1 and B0 mapping remain essential for the viable application of numerous QIBs, such as the variable flip angle framework and diffusion imaging, respectively.

Beyond the domain of MRI physics, *data harmonization* represents a post-hoc methodological approach aimed at improving intersite comparability of data-driven QIBs (Fig. 2). This strategy seeks to retrospectively mitigate variability arising from differences in acquisition protocols, scanner hardware, and reconstruction pipelines, thereby facilitating pooled analyses of large-scale multisite datasets [13]. Recently, Warrington et al. [13] recommended the combined application of (i) implicit harmonization, determining the optimal pipeline that is the most immune to the scanner effects for a given set of QIBs (referred to as image-derived phenotype by the authors) and (ii) explicit harmonization, which leverages intra-site measurements of QIBs under repeatability conditions to estimate global scaling factors that correct for the said scanner effects. In addition to providing a valuable comparison framework for harmonization methods, the authors reported comparable performance of ComBat [71] and CovBat [72] in reducing inter-scanner variability across cortical and subcortical volumes, as well as in physics-based QIBs of T2* and diffusion indices.

The ComBat has been successfully applied to enhance the statistical power of a mega-analysis using MRI data from 33 sites and 6000 subjects to distinguish healthy participants from schizophrenia patients [73]. In addition to structural and diffusion data, it was also applied to MR spectroscopy dataset, revealing so-called biological effects that would have otherwise remained hidden [74]. CovBat extends ComBat by accounting for covariance structures among harmonized features [75]. A recent CT radiomics study demonstrated that CovBat outperforms ComBat in this regard [76]. Other data harmonization approaches exist, such as RELIEF, which has been reported to surpass both ComBat and CovBat [77]. Most applications of these methods in diffusion MRI have focused on derived indices. However, De Luca et al. [78] demonstrated the feasibility of harmonizing reconstructed diffusion images directly [78].

In addition to statistics-driven harmonization methods, recent literature has seen a growing number of deep learning-based approaches, such as DeepHarmony [79] and MISPEL [80]. More recently, PhyCHarm introduced a physics-informed constraint by integrating Bloch simulations into the model [81]. For a more comprehensive analysis of data harmonization methods, the reader is referred to [75, 82–85].

## A practical guideline for streamlining portable MRI measurement pipelines

A key consideration in QIB development is balancing complexity and modularity. As summarized in Fig. 1d, modular design enables greater flexibility when each step of the measurement workflow is accessible. However, this openness comes at the cost of increased external dependencies, which can, in turn, reduce the simplicity of execution.

To address Webb’s chicken-egg dilemma, a good starting point is to *avoid putting all the eggs in one basket.* Container-mediated workflow orchestration frameworks address this problem because they simplify the management of complex computational pipelines by breaking them down into smaller, manageable tasks. One such workflow engine is Nextflow [30]: a domain-specific framework for bioinformatics, genomics, and medical imaging, designed to develop scalable, reproducible, and portable data-intensive computational pipelines.

### Basics of nextflow

Nextflow combines a declarative domain-specific language (DSL) with a reactive dataflow model. The reactive model enables Nextflow to dynamically manage data flow in response to changing data streams, eliminating the need for explicit callbacks to handle updates. For instance, a Nextflow pipeline can be linked to the raw data stream of an MRI scanner, automatically triggering reconstruction processes based on predefined patterns.

Its declarative nature enables users to define what their pipeline should achieve rather than how to execute it. Nextflow manages parallel execution, error recovery, and workflow dependencies, allowing users to focus on data transformations and file-naming conventions. This abstraction ensures seamless scalability across diverse platforms—including any operating system, cloud environments, or high-performance computing clusters—without requiring changes to the pipeline's logic.

Nextflow can be installed on any POSIX-compatible system with a single command. Pipelines are defined in scripts using Nextflow’s DSL in scripts with the.nf extension. Comprehensive documentation on DSL is available at https://nextflow.io, and https://training.nextflow.io serves as an excellent resource for getting started. For the sake of completeness, below is a high-level overview of how Nextflow pipelines are structured within an MRI-specific context:

- To build a Nextflow pipeline, users define tasks as *processes*—self-contained code blocks that specify inputs (e.g., structural MRI files like sub-*_T1w.nii.gz), outputs (e.g., defaced images such as sub-*_desc-defaced_T1w.nii.gz), and a script (e.g., *pydeface $input*). Inputs are ingested from *channels*, which dynamically match regex patterns (similar to wildcards for BIDS-structured datasets) and stream data into processes. For instance, a channel matching sub-*_T1w.nii.gz can automatically feed all participant scans into a defacing process, with outputs systematically organized into a BIDS-compliant derivatives directory.
- By default, Nextflow delegates command execution to the underlying environment, meaning external packages like PyDeface [86] must be installed and accessible (e.g., available in the system PATH for local execution). Note that alternative approaches exist to bypass local dependency management, as detailed later. Connecting an array of *processes* through *channels* in a.nf script creates a pipeline description (e.g., *my_pipeline.nf*).
- Finally, running Nextflow on a pipeline (e.g., *nextflow run my_pipeline.nf*) initiates execution of all the tasks based on the data channels that become available. Nextflow also provides interactive HTML reports, real-time tracing, directed acyclic graph (DAG) visualization, and execution logs for monitoring and debugging.
- A simple update to the pipeline configuration file, *nextflow.config*, is all it takes to switch between supported executors. For instance, setting *process.executor* = *'awsbatch'* enables job submission in the cloud without the need to manually provision or manage a virtual machine cluster.

### DSL2: a modular framework

Nextflow’s original DSL constrained pipelines to monolithic scripts, requiring error-prone code duplication or complex channel logic to reuse components. With the introduction of DSL2, Nextflow revolutionized workflow design by enabling native modularity: processes, subworkflows, and functions can now be encapsulated in separate, reusable files. This paradigm shift is particularly transformative for MRI measurement pipelines, where evolving tools and analyses demand adaptable, compartmentalized workflows.

DSL2 allows critical pipeline components—such as preprocessing steps, quality control checks, or analysis modules—to be stored in dedicated files (e.g., *modules/preprocess.nf*, *subworkflows/qc.nf*) and imported via the *include* directive. This modular architecture enforces strict input/output interfaces, clarifies data flow, and supports independent versioning of components (e.g., through GitHub or the nf-core module registry). Teams can now develop, test, and update pipeline stages in isolation before integrating them into larger workflows, streamlining collaboration.

These advancements are pivotal for MRI measurement pipelines, where evolving open-source reconstruction tools, quality control checks, and analysis steps demand adaptable, compartmentalized workflows. Unlike Snakemake [87], which relies on external templates for reuse, or Nipype and Airflow’s rigid DAGs [88, 89], DSL2 natively supports dynamic, hierarchical workflows. For a more comprehensive comparison of pipeline management systems, the reader is referred to [90].

### Key principles for optimizing nextflow in QIB development

Although most of Nextflow’s advantages come out of the box, there is a need to establish a set of principles to fully leverage its potential in QIB development:

1. Apply one process to one container mapping for modular dependency management at ease

**Fig. 3:**
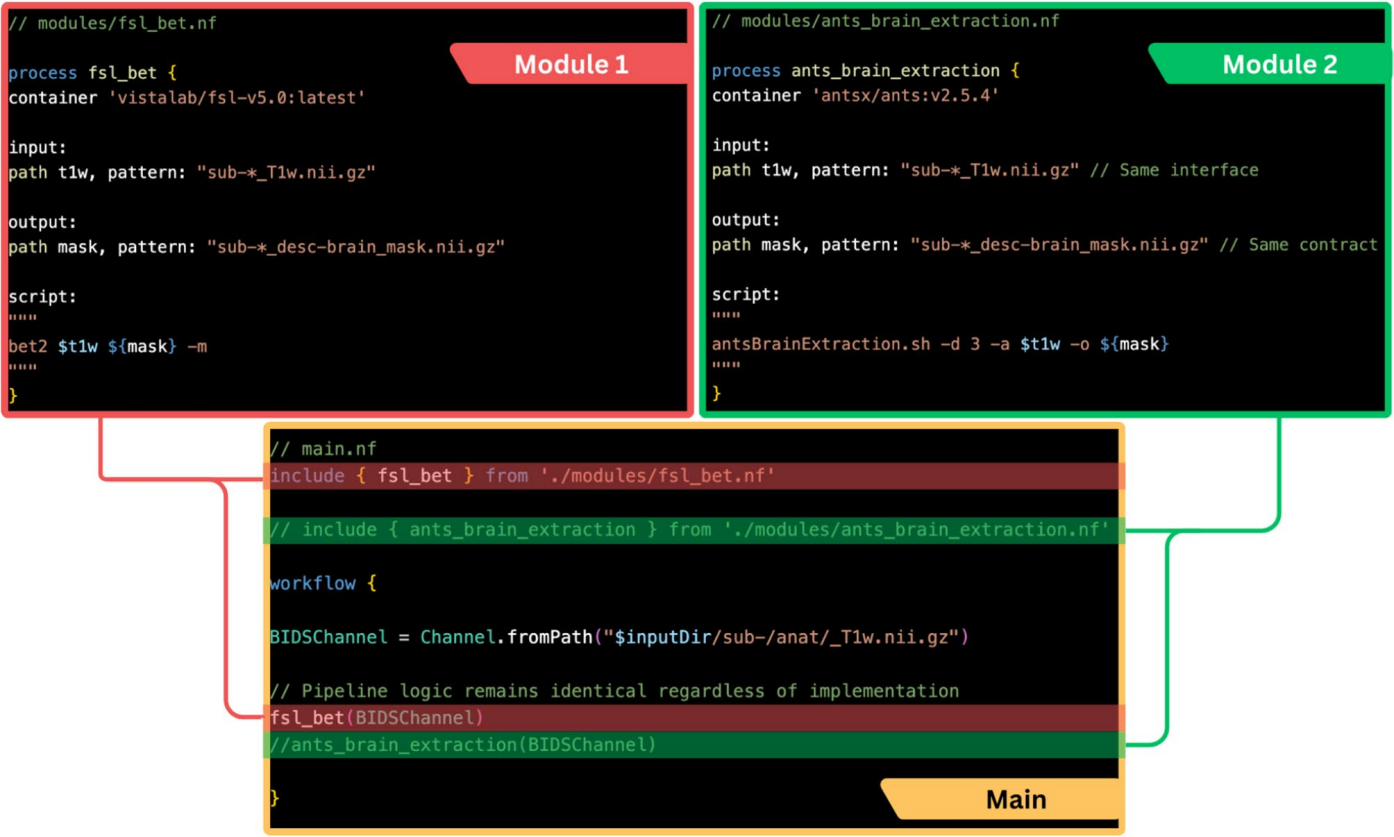
Example Nextflow snippets demonstrating how different processes—FSL (red, *fsl_bet.nf*) and ANTs (green, *ants_brain_extraction.nf*)—performing equivalent tasks through standardized input/output interfaces, enabling zero-maintenance swaps in the reconstruction pipeline (*main.nf*)

Each process in a Nextflow pipeline can be linked to a Docker or Singularity container either by specifying the container directly within the process (Fig. 3) or by defining the corresponding image name in the nextflow.config file. This enables the pipeline to execute the process within an isolated, reproducible environment that includes all necessary dependencies, in contrast to the default approach, which searches for executables in the local system environment.

When combined with the modularity introduced by DSL2, the following example outlines the high-level steps of this principle from the user’s perspective:

1. Include *motion_correction* process from ANTs sub-workflow
2. Pull *qmrlab/antsfsl* image from Docker Hub
3. Edit *nextflow.config* (or the process block) to link *motion_correction* process to the *qmrlab/antsfsl*
4. Repeat 1–3 for *wm_segment* process from FSL sub-workflow using the same container
5. Repeat 1–3 for *mt_sat* from qMRFlow using the *qmrlab/minimal* container
6. Define the dataflow between these processes to define the pipeline
7. Execute the pipeline

Instead of consolidating all these tools into a single monolithic container, the modular approach offers several advantages:

- Simplified dependency management: Users can seamlessly pull pre-configured images maintained by their developers, allowing Nextflow to orchestrate them effortlessly.
- Conflict-free dependencies: Isolating processes within dedicated containers eliminates dependency conflicts.
- Developer autonomy: Each development team can focus exclusively on their specific runtime environment.
- Avoidance of excessive wrappers: This methodology discourages the common but inefficient practice of layering academic software packages with too many wrappers.

2) Use data standards to enable declarative interlinking of the workflow processes

Nextflow’s reactive dataflow model relies on file patterns to dynamically populate channels. Therefore, community standards like ISMRM-RD [91], BIDS [92], and BIDS derivatives [93] act as structural backbones by enabling:

- Declarative routing: Naming conventions (e.g., BIDS sub-*_ses-*_T1w.nii.gz) enable automatic file discovery and channel population without hardcoded paths. For example, a motion correction process can declare it consumes *_T1w.nii.gz files, trusting they follow anatomical scan conventions.
- Interface contracts: Standards define clear input/output relationship between workflow stages. For example, a BIDS derivatives rule that segmentation masks follow *_label-WM_probseg.nii.gz lets subsequent processes reliably consume these outputs regardless of which tool generated them.

3) Swap modules, do not reinvent them

Once data standards and containerization are established, pipeline modules become interchangeable “LEGO blocks”. Figure 3 demonstrates, in DSL2, how one can easily replace one *Post* method with another due to adherence to these principles.

**Fig. 4:**
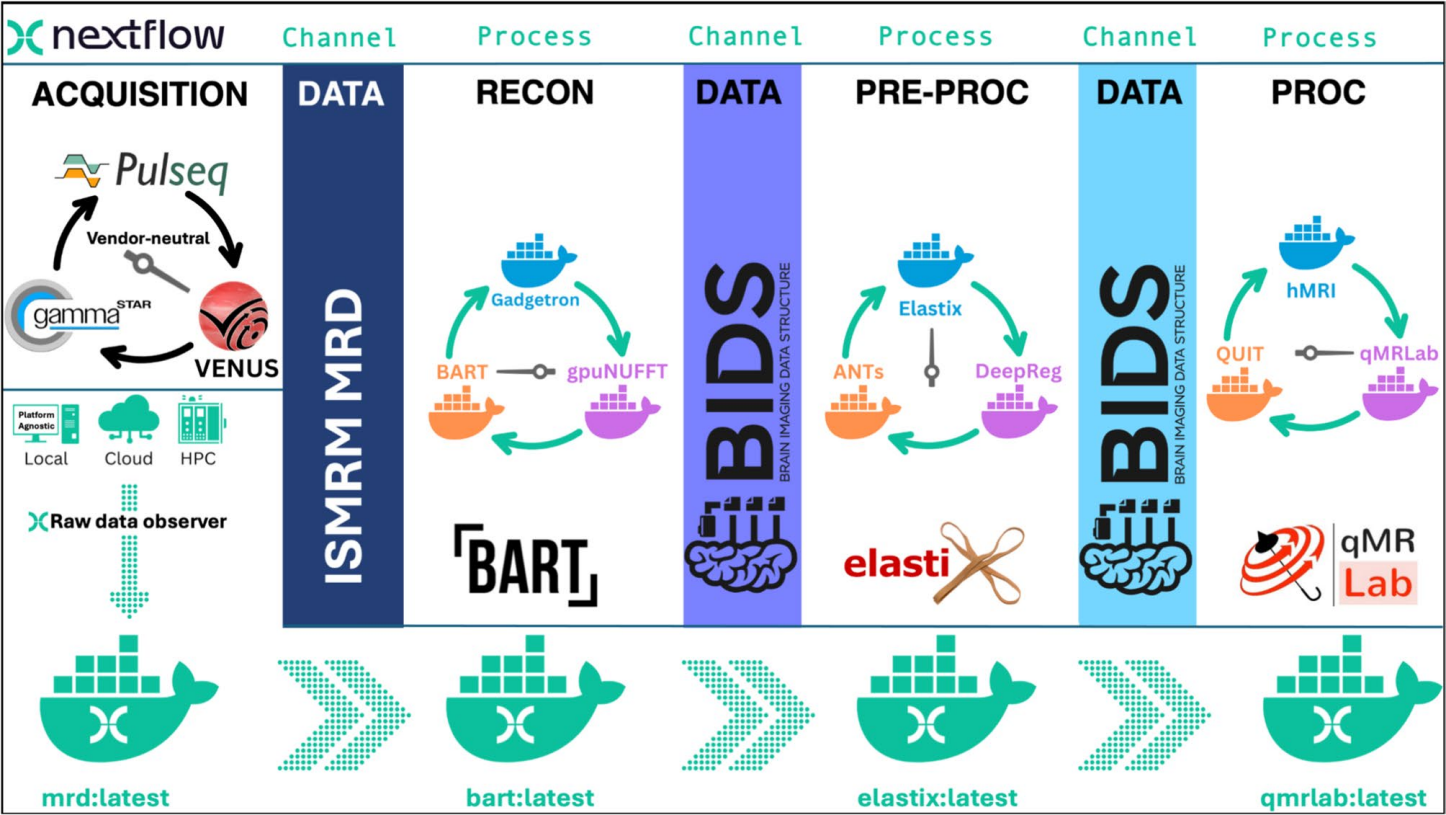
This illustration captures one of many possible (3^4^ = 81 in total) process combinations—such as Pulseq, BART, Elastix, and qMRLab—executing within their dedicated containers in isolation from each other. Each Nextflow channel's data flow is guided by established data standards and derivative conventions and the modules can be easily swapped thanks to DSL2. Platform agnostic Nextflow executors allow for running the pipeline on a local workstation, cloud computing platforms, or high-performance computing clusters

This approach can drastically reduce code complexity, particularly when the pipeline description includes all steps. Figure 4 expands on the previous DSL2 example with a schematic representation, demonstrating how numerous pipeline variations (3^4^ = 81 possible pipeline alternatives) can be generated by leveraging this easy swap feature. The ultimate vision is the creation of decentralized, portable workflows within a BIDS-apps like framework [94]. Nevertheless, unlike platforms like Brainlife [95], CBRAIN [96], or OpenNeuro [97], these workflows are designed for seamless execution across diverse infrastructures, making them essential for deployment at the point-of-care.

## Glass-box examples with qMRFlow

qMRFlow is a suite of container-mediated, data-driven and transparent qMRI pipelines, ideally starting with a vendor-neutral *Acq* [57, 58, 98]. However, when preceded by a vendor-native acquisition, the pipeline transitions from a *glass-box* to a *frosted-box* benchmark (Fig. 1c), shifting the respective strategy from *standardization* to *post-hoc workflow unification* (Fig. 2).

Powered by Nextflow, qMRFlow adapts the principles outlined in this review to enable reproducible and modular measurements. Its modules are developed to work primarily with qMRLab [30, 99], which implements a wide range of qMRI methods, including relaxometry, magnetization transfer and diffusion imaging, field mapping, and susceptibility mapping which are essential for microstructural characterization of in-vivo tissue, particularly in neurological applications.

While qMRLab offers core functionality for processing quantitative MRI data, qMRFlow enhances this by seamlessly integrating with nearly all *Recon* and *Post* software. Additionally, qMRFlow’s glass-box compatibility is a significant benefit. This compatibility was first demonstrated in [56], where qMRFlow was integrated with RTHawk [57]. The source code is available at https://github.com/qmrlab/venus, with an overview provided below:

- The *modules* folder in this repository contains several Nextflow scripts, most of which implement method-specific processes (e.g., *mt_sat.nf*) that utilize qMRLab. Additionally, *bids_patterns*.nf provides helper functions to manage BIDS-specific directory structures and rules.
- There are two main pipeline descriptions that make use of these modules: *venus-process-invivo.nf* and *venus-process-phantom.nf*. As their file names suggest, these workflows share certain processes, such as the *fitMtsat* process from *mt_sat.nf*. However, only the in-vivo pipeline imports the *generateRegionMasks* process from *ants.nf*, as this step is not required for phantom data.
- The *nextflow.config* file starts with the specification of resource allocations, including the number of CPUs, memory limits, and the maximum number of parallel runs. It also defines container configurations, linking each process to the appropriate software environment. It follows with a code block that enables the use of docker, which is then followed by process-specific parameters such as *use_b1cor* that can be changed by the users to alter the dataflow.
- Since the pipeline follows qMRI-BIDS, data channels can be structured using standardized patterns, simplifying the integration of processes and ensuring seamless workflow connectivity.

To repeat the *Recon* and *Post* steps offline, users simply provide a root folder containing multiple subject datasets, and qMRFlow automatically organizes and executes the necessary preprocessing and analysis steps. Furthermore, qMRFlow’s integration into the broader imaging neuroscience ecosystem, including connections to next-generation platforms like NeuroLibre [100], facilitates large-scale, multi-center neuroimaging studies with enhanced reproducibility and ease of use.

A more recent demonstration of qMRFlow’s glass-box compatibility is shown through its integration with Pulseq [58]. This study leveraged the open-source MPRAGE implementation provided by Pulseq to develop an end-to-end MP2RAGE [101] pipeline, deployed it to five scanners from three different vendors [102].

## Gray-box examples with tractoflow (vendor-native data)

**Fig. 5:**
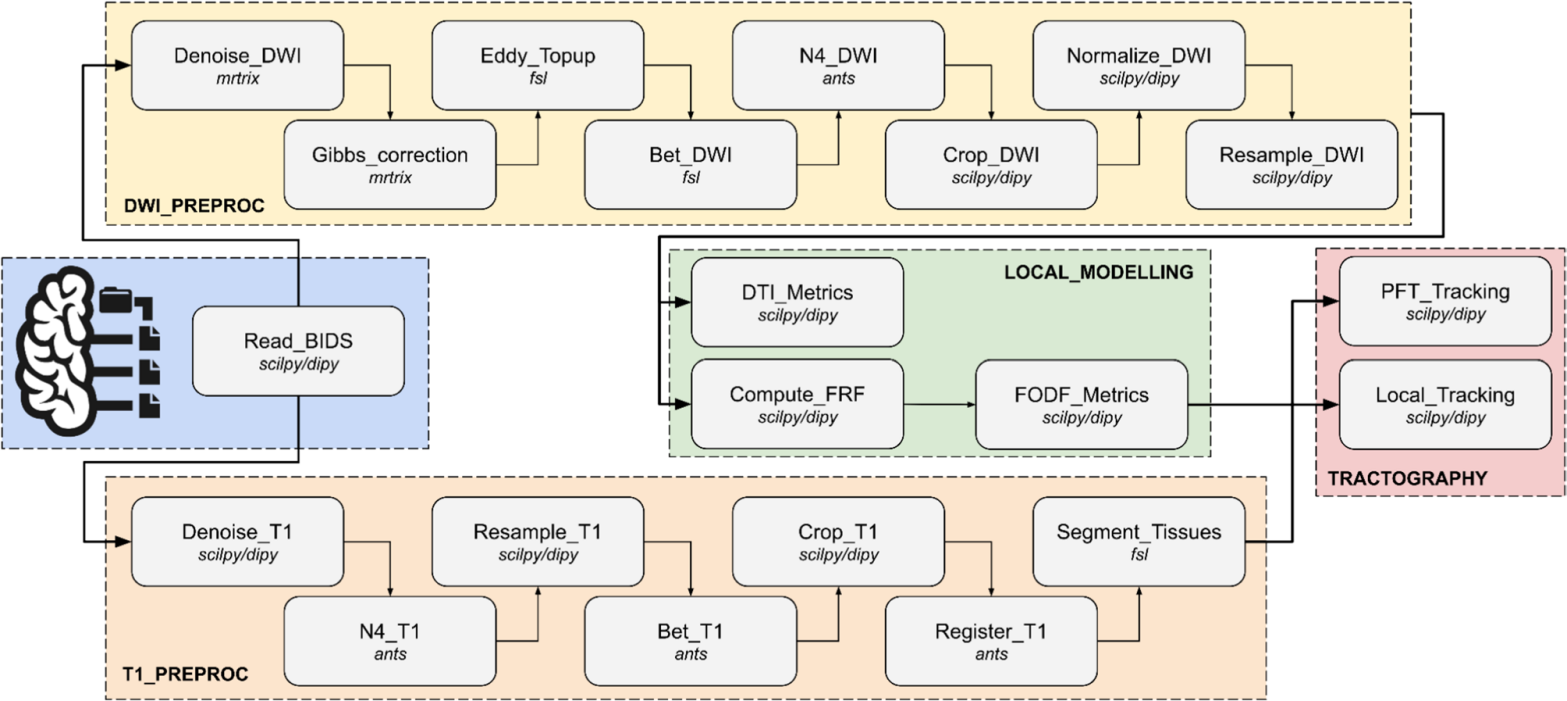
This figure represents the TractoFlow pipeline, designed as a gray-box workflow: it uses BIDS-formatted inputs from scanner reconstructions. It outputs a variety of files, ensuring reproducibility when inputs are identical. Each box in the diagram denotes a modular step, capable of saving outputs and configured for durability using dedicated Docker containers. Parameters are fully accessible and customizable through a comprehensive configuration file, allowing users to adapt the pipeline for diverse applications (e.g., healthy brain, tumor analysis, aging studies). Steps like tissue segmentation can be reconfigured to use tools like FreeSurfer, aligning with the gray-box principles

The TractoFlow pipeline processes dMRI dataset from the raw data to the tractography as a modular entity. Tractoflow was quickly adopted by researchers outside of the immediate network of developers. Powered by Nextflow DSL1 and containerization, here are the five main highlights of Tractoflow (Fig. 5):

- Efficient diffusion MRI processing pipeline from raw (i.e., vendor-reconstructed) diffusion data to tractography, which has allowed processing of the HCP [103, 104], UKBiobank [105], ADNI [36], PPMI [106], ABCD [107], and most large open-access databases by non-developers and non-experts in dMRI.
- The stochastic nature of the Tractoflow tractography pipeline makes reproducible results particularly challenging. Efforts are made to reduce variability when relaunching Tractoflow to ensure reproducible and replicable results today, tomorrow, and over time [108].
- Tractoflow provides a tradeoff between customization and curation of configuration files, making it easy-to-use for non-technical and clinician users. Profiles are provided to adapt to different use cases (aging, development, white matter hyperintensities, amongst others [108]).
- Little to no installation steps and compatibility with High-Performance Computing thanks to containers and Nextflow executors (e.g. SLURM, AWS).
- Support for multiple vendors through BIDS.

Tractoflow includes more than 20 steps that are automatically distributed depending on their dependencies and requirements and grouped into two main blocks of processes (see Fig. 3). The first is diffusion MRI pre-processing, local reconstruction of DTI and constrained spherical deconvolution (CSD), and anatomically constrained deterministic or probabilistic tractography. The second involves the T1 pre-processing and T1 analysis to register to the final diffusion MRI space and extract white matter, gray matter and CSF partial volume maps. The Tractoflow container is based on scilpy [109], dipy [110], mrtrix3 [111], ANTS [112], FSL [113] and freesurfer [114].

From the Tractoflow output tree, there are a series of optional flows available to automatically extract the main white matter bundles (rbx_flow) [115], extract NODDI (noddi_flow) [6, 116] and FreeWater (FW)-corrected DTI (FW_flow) metrics [116, 117], perform tractometry (tractometry_flow) [118], and connectomics (connectoflow) [119].

However, Tractoflow does not address completely the *gray-box principles* as stated. While the curation of configuration makes it easy to execute its workflow seamlessly, it does hinder the transparency of each process executed. In addition, each process is hardly independent from the next, from design requirements imposed by the DSL1 version of Nextflow. Its modularity at the pipeline level forces chaining to be done manually outside of the controlled environments provided by containerization and Nextflow.

Nextflow DSL2 enabled the creation of *versaFlow* [120], a modular pipeline that reaches down to the level of each process. It provides fully transparent access to all inputs, outputs and the corresponding parameters, while still curating easy-to-use configurations using Nextflow’s parameters inheritance (something that could not be done by Tractoflow because it uses a single centralized file to store this information). versaFlow can also be applied to the processing of macaque subjects, by selecting parameters and steps that optimally adapt Tractoflow, carefully validated on the PRIME-DE database [121].

## Discussion

In the section titled “Opening up the implementations, that’s how the light gets in,” we reviewed recent QIB studies that use glass-box workflows as an effective approach to tackle variability at its source, all while staying true to the first-principles foundation of MRI acquisitions. This title purposefully draws inspiration from Leonard Cohen’s famous lyrics, “There’s a crack in everything, that’s how the light gets in”. While glass-box workflows are designed for transparency, their increasing complexity introduces fractures where dependencies accumulate (Fig. 1d), particularly when interfacing with multiple vendor-specific scanner control systems.

### Are numbers extracted from MRI clinically superior to mere images?

Nearly half a century of efforts have fallen short in giving a generalizable answer to this question. MRI research silos answer it by championing a particular QIB attached to a fitting clinical use case (and even some de-facto standards) to make it reproducible across scanners [122]. Nevertheless, when we restate the same question in more practical terms—“What is to MRI what the Hounsfield Unit is to CT?”—it is difficult to provide a confident answer.

The idea that the future of MRI is quantitative emerges, fades, and resurfaces as the tagline of annual conferences on MRI methods development. Meanwhile, applied research studies leveraging MRI measurements to test biomedical hypotheses continue to proliferate, spawning new subfields and career trajectories. However, the widening gap between the ever-expanding body of rejected null hypotheses and the scarcity of QIBs that have led to changes in patient management has raised concerns among clinicians about the validity of research priorities and their practical impact on patient care.

*“Stop da music… stop da music” & “You cannot be serious”*

Opening quotes from two articles by MDs Weinberger and Radulescu [123, 124].

Following these staggering opening quotes in their articles on structural MRI, the authors warn against overinterpreting data-driven QIBs and emphasize that such findings should be framed strictly as “differences in MRI measurements” rather than definitive neurobiological conclusions such as “cortical thinning”. They further argue that the ongoing overinterpretation of confounded MRI measurements is not only problematic but also a disservice to patients and their families, potentially by misdirecting valuable taxpayer dollars—funds that could be used more effectively to improve patient care, such as by better staffing hospitals and enhancing clinical services.

 Given the unresolved translational gap, it remains too soon to determine whether numerical metrics derived from MRI offer clear clinical advantages over visual assessment alone. However, significant efforts are being made to improve the utility of QIBs, and we briefly discuss their potential benefits and challenges below.

### To standardize or to harmonize?

MRI does not directly measure anatomy; apparent tissue boundaries are influenced by systematic biases and biophysical confounders. Most data-driven QIBSs rely on these boundaries, which may reflect secondary physiological effects rather than true morphology [124]. Similarly, the “scanner (or batch) effects” linked to *Acq* are neither linear nor separable from biology [56]. Therefore, both simple transformations of QIBs into a common coordinate system—such as z-scores [125] or histogram matching [126]—and more sophisticated methods that attempt to harmonize raw scanner signals [127] remain fundamentally limited. Other limitations associated with such methods include over-harmonization, i.e., bleach-washing true biological variability, and the challenge of choosing the right technique from a plethora of harmonization options [45, 128].

Can we dispense with the differences in k-space encoding within the latent space of deep learning models? For example, CALAMITI claims to disentangle anatomy and contrast into distinct latent representations [129], achieving state-of-the-art harmonization performance. Even though these methods show promising results for improving downstream processes such as segmentation, their value in establishing reliable QIBs remains uncertain.

Simulation-based bias correction strategies targeting the opaque zone (Fig. 1a) can reduce vendor-specific differences to some extent [63]. However, some sources of variation, such as prescan calibrations, remain unaddressed. From a system theory perspective [130], it is important to note that simulation-driven corrections rely on idealized models of both the acquisition and spin systems. As a result, their effectiveness in correcting known differences between sequence implementations is constrained.

An interesting avenue for further exploration is examining inter-site effects starting from the k-space data acquired using vendor-native sequences. In the context of data-driven QIBs, variability in the reconstruction of images from multi-channel receive coils is a notable source of inconsistency [131]. For physics-driven QIBs, differences in the reconstruction of undersampled data or phase images can lead to variations in measurements [102]. A key challenge in this area is the lack of reference k-space datasets. Moreover, using synthetic k-space data (Fourier-transformed from images) as a reference has been shown to artificially improve reconstruction results [132]. It remains to be seen whether post-hoc workflow unification (Fig. 2), aligned with the frosted-box benchmark (Fig. 1b), can provide the same benefits as glass-box workflows in improving the clinical value of QIBs.

It is crucial to recognize that even the simplest QIB in MRI involves far more degrees of freedom in its calculation than the Hounsfield Unit in CT. The final form of an MR image is influenced by a complex interplay of factors, including spin dynamics, coil sensitivities, gradient nonlinearities, B0 inhomogeneities, eddy currents, susceptibility effects, RF field distortions, and physiological noise, among others. This complexity underscores the importance of returning to the first principles of MRI measurements for conscious reduction of scanner effects. We argue that taking a step back and striving for standardization in a metrological framework is a leap toward unlocking the true clinical potential of QIBs, and adopting glass-box workflows can show the way forward.

### Our pipelines can now span vendor boundaries, but can they bridge the gap between research labs and PACS workstations?

The consideration of user-friendliness is still in its early stages for deploying vendor-neutral acquisition platforms coupled with glass-box workflows. Nevertheless, exciting developments are underway both by open-source developers and vendors.

Currently, only the RTHawk proprietary platform [57] offers the flexibility to design and standardize the same user interface across vendors. It also enables the scaling of RF and gradient waveforms, as well as modifying timing parameters through the user interface. In contrast, open-source alternatives must integrate separately with each vendor's interface. While earlier versions of Pulseq did not support changing sequence parameters, the latest release (v1.5.0) has introduced support for this feature [58]. Also working towards a new major release, Gammastar provides support for changing parameters through the interface and even for AI-driven protocol optimizations [98]. Meanwhile, some major manufacturers are putting in the effort to allow easy integration of open-source *Recon *[133, 134] pipelines and even native support for interpreting Pulseq descriptions of a sequence.

The modular and portable approach to creating a comprehensive measurement pipeline presented in this review has great potential to complement these efforts, facilitate collaboration, and lower the barriers to standardizing QIB implementations. Once we standardize MRI measurement procedures through efficient use of reference objects [135, 136] and metrology principles [2], precision medicine using MRI will become more feasible and reliable [137].

## Conclusion

Unlike standardized biochemical assays which have universally accepted calibration standards across laboratories, MRI measurements remain highly variable across scanners and sites. This variability stems from the complex interplay of factors in MRI signal generation and site-specific differences in the measurement chain. Rather than constructing a Rube Goldberg machine of harmonization methods and normative modeling, we argue for returning to the first principles of MRI measurements, using standardization within a metrological framework. By embracing workflows that strive for standardization, we can bridge the gap between research innovations and clinical applications [138]. The modular and portable approach to creating QIB pipelines presented in this review offers a practical path forward, facilitating collaboration and lowering barriers to standardizing QIB implementations. This standardization, coupled with efficient use of reference objects and rigorous application of metrology principles [139], will ultimately enable more reliable and clinically meaningful QIBs, towards redefining MRI as a measurement device.
